# Predicting species distributions and community composition using satellite remote sensing predictors

**DOI:** 10.1038/s41598-021-96047-7

**Published:** 2021-08-12

**Authors:** Jesús N. Pinto-Ledezma, Jeannine Cavender-Bares

**Affiliations:** Department of Ecology, Evolution and Behavior, University of Minnesota, 1479 Gortner Ave, Saint Paul, MN 55108 USA

**Keywords:** Biodiversity, Conservation biology, Ecological modelling, Macroecology

## Abstract

Biodiversity is rapidly changing due to changes in the climate and human related activities; thus, the accurate predictions of species composition and diversity are critical to developing conservation actions and management strategies. In this paper, using satellite remote sensing products as covariates, we constructed stacked species distribution models (S-SDMs) under a Bayesian framework to build next-generation biodiversity models. Model performance of these models was assessed using oak assemblages distributed across the continental United States obtained from the National Ecological Observatory Network (NEON). This study represents an attempt to evaluate the integrated predictions of biodiversity models—including assemblage diversity and composition—obtained by stacking next-generation SDMs. We found that applying constraints to assemblage predictions, such as using the probability ranking rule, does not improve biodiversity prediction models. Furthermore, we found that independent of the stacking procedure (bS-SDM versus pS-SDM versus cS-SDM), these kinds of next-generation biodiversity models do not accurately recover the observed species composition at the plot level or ecological-community scales (NEON plots are 400 m^2^). However, these models do return reasonable predictions at macroecological scales, i.e., moderately to highly correct assignments of species identities at the scale of NEON sites (mean area ~ 27 km^2^). Our results provide insights for advancing the accuracy of prediction of assemblage diversity and composition at different spatial scales globally. An important task for future studies is to evaluate the reliability of combining S-SDMs with direct detection of species using image spectroscopy to build a new generation of biodiversity models that accurately predict and monitor ecological assemblages through time and space.

## Introduction

Species diversity and composition vary in space and time as a consequence of historical biogeographic and environmental factors and ongoing ecological processes. Since the last millennium, however, rising human population and activities have been major drivers of environmental change on Earth^1–3^. As a consequence, biodiversity is changing at a pace solely compared to the major extinction events recorded in the geologic history^1,4,5^. These rapid changes in biodiversity are impacting the capacity of ecosystems to provide services to humanity that ultimately influence our well-being^6–8^. Thus, detecting and monitoring species diversity and composition is critical to developing effective management strategies and conservation actions facing global change^3,9,10^ that move us towards international biodiversity goals, including those posed by the Intergovernmental Science-Policy Platform on Biodiversity and Ecosystem (IPBES) and the parallel United Nations’ (UN) Sustainable Development Goals (UN-SDGs)^8^ and the upcoming post-2020 Convention on Biodiversity goals.

Different approaches have been proposed for assessing and monitoring the spatial and temporal patterns of species diversity and distribution^10–12^, including biodiversity models—i.e., models that aim to predict species diversity and composition^13,14^ and remote sensing technologies and products^15–18^. Among biodiversity models, the approach of Stacked Species Distribution Models (S-SDMs) has been successfully implemented for predicting species diversity and composition^19–21^. In building S-SDMs, individual SDM are modeled as a function of environmental predictors—usually derived from interpolated climate surfaces. Subsequently, these models are stacked to produce species diversity and composition assemblage predictions^13,20^. However, despite successful S-SDMs predictions, most previous studies were restricted to small geographic areas (~ 700 km^2^)^19,20,22^, limiting the potential of these models for biodiversity prediction and monitoring over large geographic areas, including at continental scales. Moreover, the uncertainty associated with the underlying data (i.e., occurrence records and environmental covariates) can negatively impact species model predictions^23,24^ and consequently the reliability of these kinds of biodiversity models.

Remote sensing products (RS-products) have been increasingly used to derive metrics that allow tracking biodiversity from space^18,25,26^, monitoring the state of human impacts^3,27^, as predictors for describing large patterns of species diversity^28–30^ or to derive Essential Biodiversity Variables, i.e., measures that allow the detection and quantification of biodiversity changes^12,31,32^. Despite their high spatial and temporal resolution, quasi-global coverage and range of data products (e.g., precipitation, plant productivity, biophysical variables, land cover), RS-products have been rarely used as predictors for biodiversity models^17,30,33^. RS-products have been dubbed important “next-generation” environmental predictors in biodiversity models^34^, given that remote sensing continuously captures an increasing range of Earth’s biophysical features at global scale^35,36^, avoiding the uncertainty associated with environmental predictors derived from traditional climatic data. Furthermore, species models derived from RS-products perform as well as those derived from interpolated climate surfaces and have the potential to provide predictions with greater spatial resolution^30^.

A crucial challenge for detecting and monitoring biodiversity is the integration of modeling approaches with RS-products in order to build next-generation biodiversity models at different spatial and temporal scale and levels of organization^17,34^. In addition, recent progress has been made in developing methods for use in S-SDM construction as well as in their evaluations^21,37^. By combining advances in S-SD modeling with RS-products, these developments could overcome some of the issues faced by traditional stacked models in predicting species richness and assemblage composition at different spatial scales. Therefore, the general aim of this paper is twofold: (1) to combine S-SDMs and RS-products in order to build next-generation biodiversity models, and (2) to perform a rigorous evaluation of biodiversity model predictions. In doing so, we applied a sequential procedure that allows the prediction and evaluation of species assemblage diversity and composition at continental scale. This procedure was implemented using the oak clade (genus *Quercus*) as a model study within the geographical area corresponding to the conterminous United States. We focused on this clade because oaks are widely distributed forest dominants, and they have the highest species richness and highest total biomass of any woody group in the conterminous U.S.^38^. Nevertheless, individual species have contrasting environmental distributions and range sizes. These attributes make the oaks both an important study system and a useful one for testing next generation SDMs.

Specifically, we begin by constructing individual species distribution models for each oak species using RS-products as covariates. The models we developed are designed to be biologically meaningful—i.e., we selected environmental covariates derived from Earth observations that are relevant to explaining distributions of trees in the oak genus—and to account for the accessible area of each species. To accomplish the latter, we constrained the training and transference areas. These models were then stacked to obtain predictions of oak species assemblage diversity and composition using different stacking procedures. Next, we conducted a comprehensive evaluation of assemblage predictions using observed assemblages’ diversity and composition obtained from the National Ecological Observatory Network (NEON).

## Results

### Species model predictions

**Figure 1:**
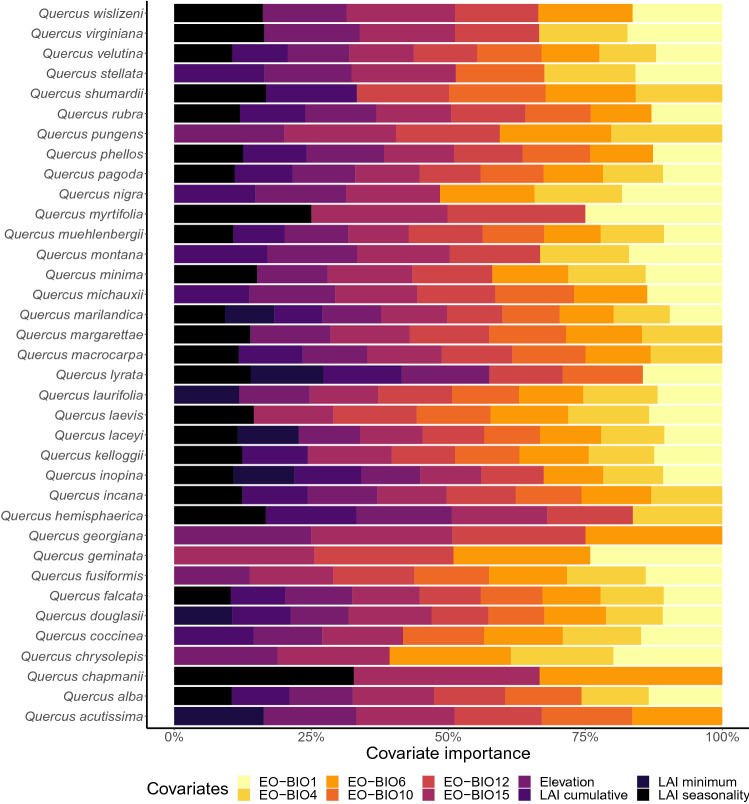
Covariate importance or the relative contributions (%) of the 10 covariates used for modeling the potential distribution of oak species in the Continental United States.

Oak species predictions were successfully calibrated for all species (Supplementary Table S1), showing high prediction accuracy as measured by the AUC (area under the receiver operating characteristic curve) and the True skill statistic (TSS) metrics (median ± SD: AUC 0.96 ± 0.03, TSS 0.82 ± 0.09; Supplementary Table S1). The number of covariates that better explain the probability of occurrence of oak species varies from 3 (*Q. chapmanii*; AUC = 0.99, TSS = 0.95) to 10 (*Q. marilandica*; AUC = 0.95, TSS = 0.76). Covariate importance evaluation (Fig. 1) indicate that precipitation seasonality (EO-BIO15 = 13.5%), annual precipitation (EO-BIO12 = 12.4%) and elevation (Elevation = 12%) were the covariates that contributed most to predict the occurrence of oak species; nonetheless, the number of covariates and their percentage of contribution varies considerably per species (Fig. 1). Notably, biophysical covariates such as LAI cumulative and LAI seasonality were selected as important covariates in predicting the occurrence for 21 and 23 oak species (Fig. 1), respectively.

**Figure 2:**
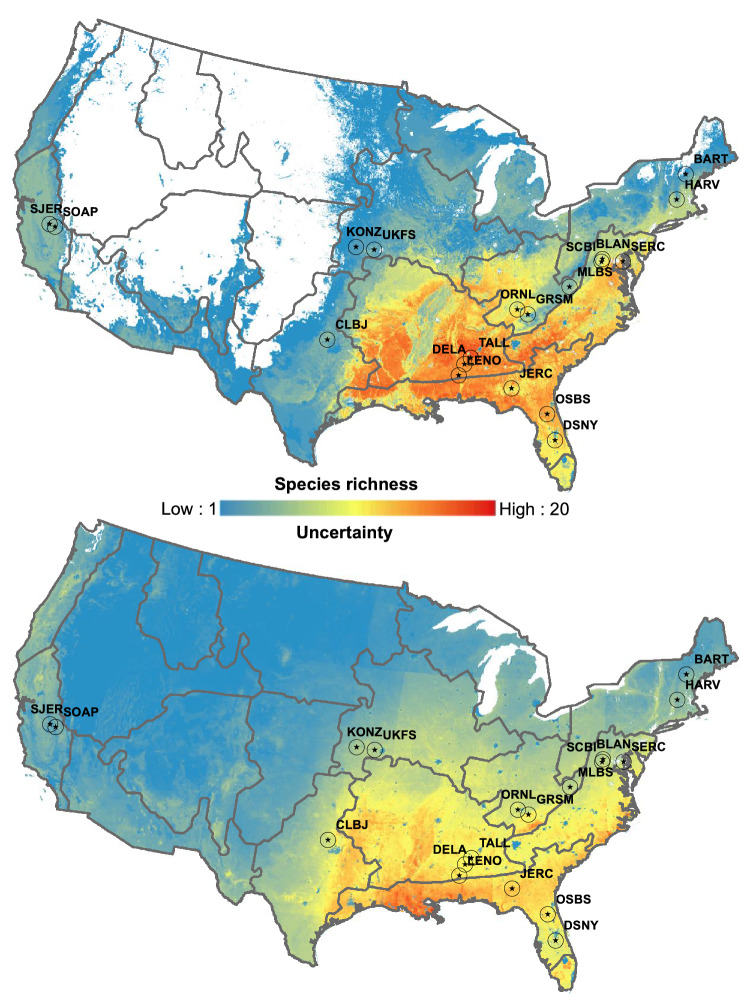
Macroecological patterns of the number co-occurring oak species (top panel) and uncertainty (low panel) estimated by stacking individual species distribution model binary predictions (S-SDMs). Numbers on the legend represent the number of co-occurring within each pixel. In addition, blue and red colors mean low and high species co-occurrence (uncertainty), respectively. Star symbols (and acronyms) on the map represent the nineteen NSF-NEON sites that have data of oak species assemblages. Overlaid gray polygons represent the NSF-NEON ecoclimatic domains. Map projection: Lambert Azimuthal Equal Area. Maps were generated using ArcGis Desktop 10.6 (https://desktop.arcgis.com/).

Macroecological patterns of oak species richness indicate that oak species are distributed unevenly, showing high concentration in the Southeast United States, specifically in the NEON domains of the Ozark complex, the Southeast and the Mid-Atlantic (Fig. 2 top panel). Uncertainty predictions indicate that the Southeast NEON domain is the region with major uncertainty (Fig. 2 bottom panel).

### Assemblage predictions

**Figure 3:**
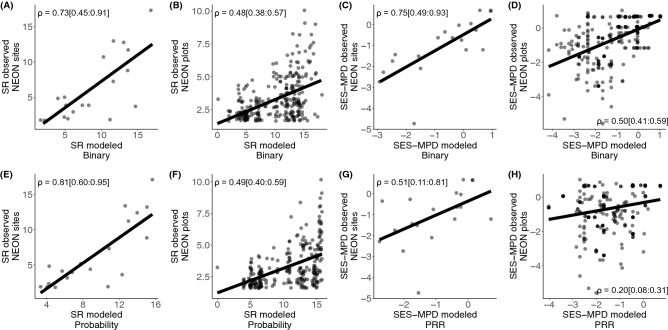
Predicted versus observed number of co-occurring oak species (left panels) and phylogenetic assemblage structure (right panels) at site and plot scales. Rho (ρ) values within each panel represent the median Pearson’s correlation coefficients estimated from posterior distribution and the 95% of credible intervals within brackets.

Mean observed richness per assemblage at the plot scale (i.e., NEON plots of 400 m^2^) was 3.27 (SD = 1.59, max = 10, min = 2), and mean richness prediction per assemblage for bS-SDM_PLOT_ was 10.09 (SD = 4.19, max = 17, min = 1) and 10.54 (SD = 4.08, max = 15.80, min = 0) for pS-SDM_PLOT_. Mean observed richness at the site scale (i.e., NEON sites; mean area ~ 27 km^2^) was 6.37 (SD = 4.68, max = 17, min = 2), while the prediction for bS-SDM_SITE_ was 8.79 (SD = 4.65, max = 17, min = 2) and for pS-SDM_SITE_ was 9.23 (SD = 4.29, max = 15.64, min = 3.33). Bayesian correlations revealed mid positive correlations between the observed and the predicted richness derived from both the bS-SDM_PLOT_ (ρ = 0.48 [0.38:0.57, 95% credible interval (CI)], Fig. 3B) and pS-SDM_PLOT_ (ρ = 0.49 [0.40:0.59, 95% credible interval (CI)], Fig. 3F) at the plot scale. At the site scale the correlation increased for both bS-SDM_SITE_ (ρ = 0.73 [0.45:0.91], Fig. 3A) and pS-SDM_SITE_ (ρ = 0.81 [0.60:0.95], Fig. 3E). Although, richness predictions under pS-SDM correlate better with the observed richness, no evidence was found in favor of pS-SDM over bS-SDM (MAP_SITE_ = 0.46, ROPE_SITE_ = 48%; MAP_PLOT_ = 0, ROPE_PLOT_ = 27%).

**Figure 4:**
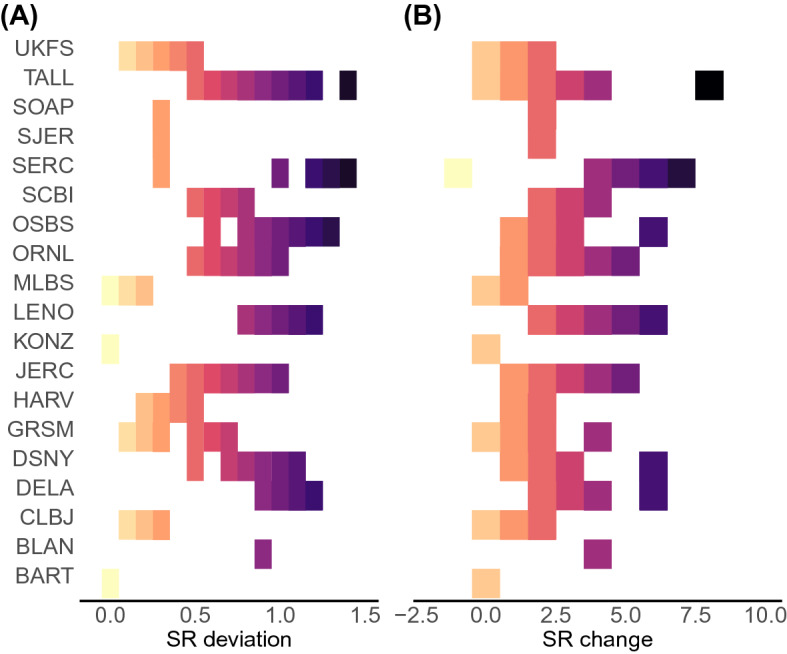
Accuracy of the number of co-occurring oak species predictions at site and plot scale. (**A**) Species richness deviation, measured as the absolute deviation in assemblage richness predictions divided by the maximum observed NEON plot species richness, where low and high values represent high and low accuracy respectively. (**B**) Species richness change, that represent the species loss or gain at plot scale. Negative values represent the number of species lost (underprediction) while positive values the gain of species (overprediction).

Mean absolute deviation between observed species richness and species richness predictions derived from the bS-SDM was 0.68 (SD = 0.37) and 0.16 (SD = 0.18) for the plot and site scales, respectively (Fig. 4A). The mean absolute deviation for pS-SDM was 0.73 (SD = 0.35) at the local scale and 0.18 (SD = 0.14) at the site scale. The mean species richness change (Fig. 4B) at the plot scale was 2.4 (SD = 1.69) for bS-SDM and 2.56 (SD = 1.65), while at the site scale it was 0.75 (SD = 1.18) and 0.85 (SD = 1.03) for bS-SDM and pS-SDM, respectively. Bayesian-ANOVAs reveled that bS-SDM outperformed pS-SDM, in predicting species richness, i.e., show low absolute deviation at the plot scale (MAP_PLOT_ = 0) but not at the site scale (MAP_SITE_ = 0.43). Similar results were found for species richness change metric (MAP_PLOT_ = 0, MAP_SITE_ = 0.59).

**Figure 5:**
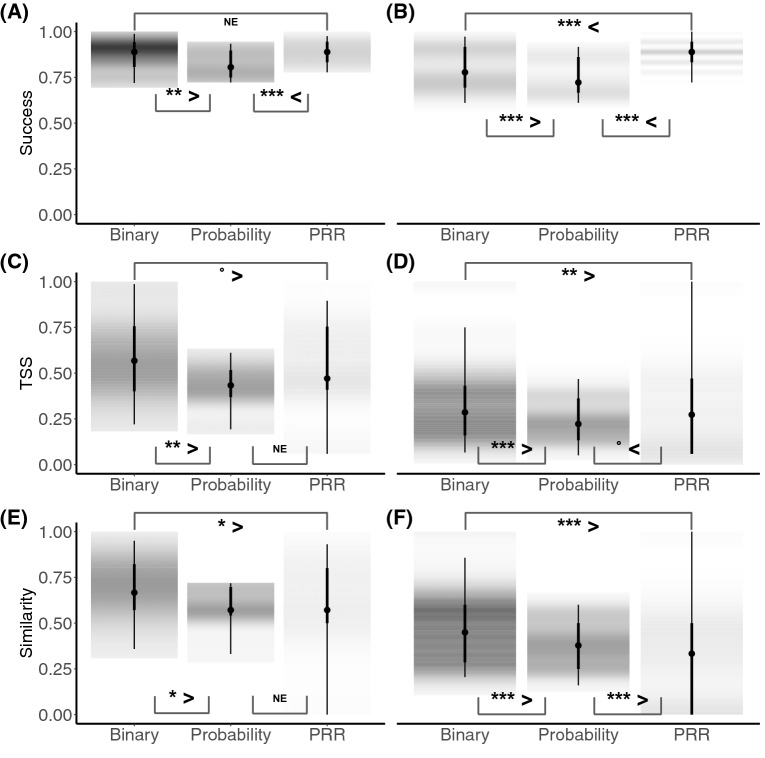
Accuracy of assemblage composition predictions at site and plot scales. Left panels (**A**,**C**,**E**) metrics at site scales and right panels (**B**,**D**,**E**) metrics at plot scale. Significant differences between stacking procedures (bS-SDM versus pS-SDM versus cS-SDM) were evaluated using Bayesian ANOVAs with NEON plot IDs as random effect (significance levels for the Maximum A Posteriori p-values are: ***p = 0, **p < 0.01, *p < 0.05, •p < 0.1, *NE* no evidence).

Mean assemblage prediction success was high for the three stacking procedures at both the site scale (bS-SDM = 0.79 ± 0.08, pS-SDM = 0.75 ± 0.07, cS-SDM = 0.88 ± 0.06; Fig. 5A) and the plot scale (bS-SDM = 0.87 ± 0.11, pS-SDM = 0.82 ± 0.10, cS-SDM = 0.88 ± 0.07; Fig. 5B). Assemblage TSS across all plots and sites ranged from low to high for the three stacking procedures (Fig. 5C,D). Mean assemblage similarity estimated using the Sørensen index were mid-low at plot scale (bS-SDM = 0.47 ± 0.17, pS-SDM = 0.39 ± 0.12, cS-SDM = 0.30 ± 0.28; Fig. 5E) while mid-high at the site scale (bS-SDM = 0.69 ± 0.17, pS-SDM = 0.59 ± 0.11, cS-SDM = 0.58 ± 0.27; Fig. 5F). Furthermore, although the mean values of the metrics used to evaluate the accuracy in assemblage predictions were relatively similar between the three stacking procedures (Fig. 5), Bayesian-ANOVAs revealed strong evidence in favor of the binary stacking (bS-SDM) in predicting assemblage composition, in other words, bS-SDM outperformed pS-SDM and cS-SDM. The higher performance of the bS-SDM procedure is consistent at both site and local scales, with the exception of prediction success metric, where cS-SDM outperformed bS-SDM at the plot scale (Fig. 5B) and no evidence was found at the site scale (Fig. 5A).

### Phylogenetic assemblage structure

We were also interested in evaluating whether species composition of the assemblage predictions achieved by stacking SDMs returned similar patterns of phylogenetic structure as the observed assemblages. We found that the average SES-MPD at the plot and site scales for the observed assemblages were as follows: NEON_PLOT_ = − 0.66 ± 1.30 and NEON_SITE_ = − 1.04 ± 1.34; bS-SDM_PLOT_ = − 1.12 ± 1.23 and bS-SDM_SITE_ = − 0.71 ± 1.3; cS-SDM_PLOT_ = − 1.42 ± 1.09 and cS-SDM_SITE_ = − 1.02 ± 1.03. In addition, most of the predicted assemblages presented negative SES-MPD values (right panels in Fig. 3), indicating that the dominant pattern of phylogenetic structure in the predicted oak assemblages is overdispersion, a pattern that is commonly observed in natural oak assemblages^39,40^. Bayesian correlations showed mid to high positive associations between the phylogenetic structure obtained from the observed (NEON) and the bS-SDM at plot (ρ = 0.50 [0.41:0.59], Fig. 3D) and site (ρ = 0.75 [0.49:0.93], Fig. 3C) scales, respectively. The phylogenetic structure derived from the cS-SDM procedure, despite showing positive associations with the observed values, were mid and low for cS-SDM_SITE_ (ρ = 0.51 [0.11:0.81], Fig. 3G) and cS-SDM_PLOT_ (ρ = 0.20 [0.08:0.31], Fig. 3H), respectively.

## Discussion

The use of RS-products in biodiversity models has been hailed as a transformative approach for providing simple and flexible biodiversity predictions^17,34^. In this study we evaluated the reliability of stacked species distribution models (S-SDM) to predict biodiversity in terms of species richness and composition within local assemblages^13^ based on individual SDMs derived only from RS-products. Our results indicate that the combination of S-SDM and RS-products perform well in predicting plot-level biodiversity as assessed by several important metrics. However, when we compared the S-SDM predictions of phylogenetic structure of oak assemblages at the plot-level with the observed structure (measured in terms of species richness and mean phylogenetic distance), the predictions tended to overestimate the diversity, even though the predicted and observed metrics were positively correlated. This result is consistent using the stacking procedure. We also found that when oak assemblages are evaluated at large scales, i.e., at the site scale, S-SDM recovered the observed pattern well, i.e., patterns of predicted diversity were highly similar to those observed at the NEON sites (Figs. 3A,C, 5A,C,E). Consequently, our results show that biodiversity models in combination with RS-products do not necessarily predict biodiversity well at the plot-level scale, but they do well at larger scales. In other words, the S-SDMs that use RS-products as environmental predictors are very useful for detecting macroecological patterns of biodiversity. These results spur new insights for biodiversity prediction and monitoring at different spatial scales.

Biodiversity models, such as the S-SDMs, depend on the reliability of individual species models (SDM)^14^. SDMs represent abstractions of species Hutchinsonian niches, in which species distributions are constrained by both the abiotic environment and biotic interactions with other species (Hutchinson's duality sensu^41^ see also Refs.^30,42^). Our results show that individual oak SDMs constructed using environmental covariates derived from RS-products, have good accuracy (Supplementary Table S1). However, by stacking a suite of SDMs to obtain assemblage composition predictions, no biotic constraints are considered. Consequently, S-SDM predictions tend to overestimate the number of species within local assemblages^11,14^. We found that species richness predictions using S-SDM overpredict the number of oak species that are observed in naturally assembled oak communities (Fig. 4A,B). This might be related to the proportion of common (high prevalence) and rare (low prevalence) species that are co-occurring in these assemblages, that in turn is affected by microhabitat variables that allow oak species to differentiate locally. Indeed, soil moisture (measured in 20 × 50 m plots on the ground) explain oak species trait distributions better than climate variables^43^. Local fire dynamics, not predicted by climate variables, may additionally be important in limiting and driving local species distributions^44,45^.

In addition, it has been suggested that incorporating some ecological assembly rules, e.g., the probability ranking rule approach (PRR), in S-SDM predictions can minimize assemblage overpredictions^14,19,46^. Our results corroborate this assumption; specifically, predicting species assemblages by simply overlaying individual SDMs, tends to overpredict the number of species present in local assemblages, especially at the scale of plots (Fig. 3B,F). However, through the implementation of PRR in S-SDM predictions—i.e., by constraining the number of species present within local assemblages^14^—we found that cS-SDM do not outperform bS-SDM assemblage predictions at both site and plot scales (Fig. 5). Rather, we found that simple stacking procedures (e.g., bS-SDM) outperform cS-SDM when the goal is to evaluate assemblage species composition (Fig. 5C–F). This conclusion is corroborated by the observation of low correlation between the phylogenetic structure of naturally assembled oaks at NEON sites and plots and assemblages predicted using cS-SDM (NEON sites = 0.51[0.11:0.81, 95% credible interval (CI)] and NEON plots = 0.20[0.08:0.31], see also Fig. 3G,H).

While applying constraints to assemblage predictions may be advantageous for predicting species richness^11,14^, this is not the case for predicting species identities or composition within assemblages, particularly at finer scales. Indeed, we found that no stacking procedure accurately recovered the species identities at the plot scale (Fig. 5D,F). Not surprisingly, predictions of assemblage richness and composition improved dramatically with increasing scale (Fig. 3A–E, 5C–E), a pattern observed independently of the implementation of PRR (cS-SDM) or its non-implementation (bS-SDM and pS-SDM). These contrasting results might be a consequence of the fact that S-SDMs were primarily designed for the description and assessment of macroecological patterns of species diversity^11,13^. This scale dependence might suggest that at larger spatial scales more uncertainty in model predictions is allowed, in other words, the probability of adding species in assemblages by increasing grain size also increases^47^ as well as the randomness in co-occurrence patterns^40,48^. This is not a small issue as no species occur within their complete geographic range, i.e., there are discontinuities in the species occurrence patterns that can be detected only at local or ecological scales. In addition, although RS-products are useful covariates for the spatial modelling of biodiversity, that allow reducing the uncertainty associated with environmental covariates in model predictions^30^, the coarse grain of current RS-products hampers accurate predictions of biodiversity at ecological scales^30,36,49^. In other words, the broad spatial resolution of current RS-products (usually with a pixel size of 30 arc-seconds or ~ 1 km) limits our ability to capture environmental features at ecological scales (e.g., microtopography, soil moisture, landscape structure) that ultimately determine the coexistence of species locally.

Furthermore, we acknowledge that we evaluated only one type of biodiversity model—S-SDM using two stacking procedures—and that other biodiversity models such as the Joint-SDM (J-SDM)^50,51^, could potentially improve biodiversity predictions by jointly estimating both the species-environment relationships (as in SDMs) and the species-pairwise dependencies—that reflect patterns of co-occurrence—not accounted by the covariates^51–53^. Nevertheless, recent studies have suggested that J-SDM do not improve species assemblage predictions^21,53^, in fact, richness predictions from S-SDMs and J-SDMs tend to return similar outcomes^53^, but see Ref.^54^. Although J-SDM represent an outstanding alternative for modeling and predicting biodiversity at assemblage level^51,54^, this kind of biodiversity models become untraceable when they are applied to large datasets^53,55^ (e.g., NEON or FIA datasets), in other words, the species-pairwise dependencies matrix or residual correlation matrix increases quadratically by adding new species to the dataset^55^. Further research is needed to correctly predict biodiversity at the assemblage level.

The evaluation presented here is meant to stimulate further methodological and empirical research to better predict biodiversity at different spatial and temporal scales and levels of organization. A promising approach for this purpose is the hierarchical modeling of species distributions (H-SDMs)^56,57^. H-SDMs allow the simultaneous modeling of spatial patterns of biodiversity at ecological and regional scales. In constructing H-SDMs, individual SDMs are first fit at regional and landscape scales, and the two predictions are fused to obtain a single species SDM, i.e., the regional model is rescaled to the landscape model^56,57^. This is an interesting approach because it fuses the benefits of macroecological covariates (derived from climatic surfaces or RS-products) with those derived from high-resolution remote sensing products^56^.

An unparalleled alternative is the direct detection of species using imaging spectroscopy^58,59^. For example, leaf-spectra variation among individual plants obtained using imaging spectroscopy provide sufficient information for the correct assignment of populations to species to clades^60,61^ and airborne imagery accurately assigns vegetation canopies to species^62^. Current and forthcoming hyperspectral images from DESIS sensors and forthcoming SBG and CHIMES sensors, among others^18,63,64^ will capture information from the Earth at fine spectral resolution, allowing the estimation of plant traits, plant nutrient content, biophysical variables (e.g., leaf area index, biomass), that can be used for direct detection of functional and perhaps community diversity from space^36^. Combining the detection of species using imaging spectroscopy with SDMs to build a new generation of biodiversity models, may open new avenues for the accurately assignment of species and assessment of ecological assemblages through space and time^17,61,65,66^, a critical hurdle to overcome in addressing the challenges posed by the global change.

## Conclusion

Recent review papers^17,34^ proposed the applicability of remote sensing data as environmental covariates in the construction of next-generation SDMs. Here using environmental covariates derived completely from remote sensing products, we modelled the distribution of oaks (genus *Quercus*) and predicted the number and composition of species within assemblages at different grain sizes. Despite high variability in the predictions, modeled oak assemblages showed phylogenetic overdispersion, indicating that models recovered the observed pattern of distantly related oak species co-occurring more often than expected. Overall, we conclude that species richness can be predicted with high accuracy by applying constraints to the predictions. However, accurate predictions of species identities are still an evolving task. We suggest two alternatives (i.e., H-SDMs and the direct detection of species using imaging spectroscopy) that might increase the accuracy in assemblage composition predictions, hence, future studies should evaluate the reliability of these two alternatives using different taxa and across geographical settings.

## Methods

### Species occurrence dataset

The genus *Quercus* includes 91 species widely distributed across U.S. and showing a marked longitudinal species diversity gradient, with high species concentration in south-eastern North America. Our main occurrence dataset was assembled in a previous study^67^. We completed the main dataset using the Integrated Digitized Biocollections (iDigBio) and the Global Biodiversity Information Facility (GBIF) (data downloaded between 15 and 18 December, 2020) and collections from the second author (JCB)^68^. All occurrence data were visually examined and any localities that were outside the known geographical range of the species, in unrealistic locations (e.g., water bodies, crop fields) or in botanical gardens were discarded for accuracy. In addition, to avoid problems of spatial sampling bias and spatial autocorrelation we thinned the occurrence records of each species using a spatial thinning algorithm^69^ with thinning distance of 1 km for species with less than 100 occurrences up to 5 km for species with more than 10,000 records.

### Species and assemblage composition evaluation data

Ecological niche models represent abstractions of the environmentally suitable areas for species to maintain long-term viable populations^42^. The presence of a species in a locality or a grid cell informs us about the areas that are environmentally suitable for that species^42,70^. Its absences inform us of the opposite pattern—namely those areas that are either not environmentally suitable for the species or are the result of historical contingences, biotic interactions that constraint the species presence even if the physical environment is suitable, recent extirpation events such as those caused by land use change, or simply because the species was not detected^42,70^. Here, using an independent dataset of true presences and absences from NEON^71^, we evaluate the assemblages’ diversity and composition predictions. NEON data follows a nested structure in which subplots are nested in larger plots and these plots in turn are nested within large areas or NEON Sites. More specifically, the presence of species is recorded at 10- and 100-m^2^ subplots. The recorded species within the subplots are then combined to obtain a complete species list for a plot of 400-m^2^^71^. To obtain a complete list of oak species for each NEON site, we combined the species lists from the plots embedded in each site.

Here we used the species presence and absence at plot and site scale for assemblage predictions. This dataset includes a total of 277 plots of 400-m^2^ embedded in 19 NEON sites, spanning 8 out of the 17 NEON ecoclimatic domains in the continental United States. In addition, 36 of the 91 oak species were found in the NEON dataset; thus, we restricted all our analyses to this set of species (Fig. 1).

### Environmental data derived from remote sensing

Remote sensing products used as environmental inputs to our models included a set of covariates that allow the description of distribution and environments occupied by oak species (EO-bioclimatic covariates) and covariates that allow the discrimination of local features not captured by bioclimatic information (biophysical covariates)^30^. Bioclimatic covariates were constructed by combining monthly Land Surface Temperature and Emissivity (LSTE) from MODIS (MOD11C3) and monthly precipitation from Climate Hazards group Infrared Precipitation with Stations (CHIRPS) following Deblauwe et al.^72^. Specifically, monthly LSTE and CHRIPS data were used as input to construct 19 bioclimatic covariates based on Earth observations^72^, similar to those of WorldClim, using the function ‘biovars’ in the R package dismo^73^. Prior to EO-bioclimatic construction global monthly LSTE (2001–2019) and CHIRPS (1981–2019) were downloaded using the interfaces EOSDIS Earthdata (https://earthdata.nasa.gov/) and the Climate Hazards Center (https://data.chc.ucsb.edu/products/CHIRPS-2.0/), respectively. We named each bioclimatic variable derived from Earth observation as EO-bioclimatic variables (e.g., EO-BIO1 or EO-MAT, EO-BIO12 or EO-AP) in order to avoid confusion with those bioclimatic variables derived from interpolated climate surfaces. EO-bioclimatic covariates used or modeling oak distributions include mean annual temperature (EO-BIO1), temperature seasonality (EO-BIO4), minimum temperature of coldest month (EO-BIO6), mean temperature of warmest quarter (EO-BIO10), annual precipitation (EO-BIO12), and precipitation seasonality (EO-BIO15), all critical for the distribution and differentiation of oak species^67,68^.

Biophysical covariates include Leaf Area Index (LAI) composites (cumulative, minimum and seasonality)^28–30^. LAI data were obtained from MODIS Terra/Aqua MOD15A2 over a 15-year period (2001–2015) using the interface EOSDIS Earthdata. The 15-year LAI composite provides a representation of the spatial variation of biophysical variables of different components of vegetation and ecosystems over the course of this time frame^28,30^. LAI strongly co-varies with the physical environment; for instance, higher LAI is associated with warmer, wetter and stable environments, whereas lower LAI with cooler, drier and less stable environments^74–76^. In particular, LAI has a strong relationship with climate and captures the dynamics of the growing season, including vegetation seasonality—important for characterizing plant species geographical ranges^30,35^. We posit, therefore, that the spatial and temporal variation in LAI—half of the total green leaf area per unit of horizontal ground surface area^77^—represents an important variable for determining species distributions that integrates across biotic and abiotic factors^42,78^. Finally, we also included mean elevation or altitude from Shuttle Radar Topography Mission (SRTM). SRTM is a high-resolution digital elevation model of the Earth that has been used for mapping and monitoring the earth’s surface^79^ and an important variable for predicting species distributions and community composition^20,80^.

Given that the original pixel size of both LSTE and CHIRPS products is 0.05° (5.6 km at the equator), we downscaled these products to 1 km to match the spatial resolution of both, biophysical covariates and SRTM. We examined the match between the original and the downscaled EO-bioclimatic covariates using Spearman rank correlations (ρ) and the Schoener’s D metric^81^ that is commonly used for estimating environmental similarity. D metric ranges from 0 (no similarity) to 1 (complete similarity). If the values of ρ and D are close to 1, we can hypothesize that exist a high correspondence between the two sets of EO-bioclimatic covariates, and consequently the downscaling process has null or neglected effect on the species distribution models. These analyses were performed for each species (N = 36) separately (Supplementary Table S2). We found high correspondence between the two data sets of EO-bioclimatic covariates (mean ± SD of ρ across species = 0.985 ± 0.015 and mean ± SD of D across species = 0.994 ± 0.003), thus we can conclude that our SDMs using downscaled EO-bioclimatic covariates are not compromised (Supplementary Table S2).

### Modeling framework

To obtain reliable SDM predictions it is necessary to define the accessible area for a species in geographical space, i.e., the area that has been accessible to the species within a given period of time^42,82^. We defined the accessible area for each oak species as a bounding box around the known species occurrences, i.e., the occurrence records from the occurrence dataset, plus ~ 300 km beyond each bound. This procedure accounts for approximate species dispersal within a geographical domain and has been shown to improve model performance^82,83^. In other words, this procedure provides a conservative spatial representation of the environmental space in which a given species has potentially dispersed and is detectable. The individual accessible areas or species-specific accessible areas were then used to mask the covariates for each oak species and to constraint the random generation of pseudoabsences or background points before modeling species potential distributions. The number of pseudoabsences generated was similar to the number of presences^84,85^.

We modeled species potential distributions using Bayesian additive regression trees (BART)^86^. BART is a classification tree method defined by a prior distribution and a likelihood for returning occurrence predictions that enables the quantification of uncertainty around the predictions and the estimation of the marginal effects of the covariates^86–88^. BART models were run with the default parameters as implemented in dbarts^89^ through the R package embarcadero^88^. More specifically, BART models were run using 200 trees and 1000 back-fitting Marcov Chain Monte Carlo (MCMC)^90^ iterations, discarding 20% as burn-ins. Model performance or predictive ability was evaluated using two measures, the area under the receiver operating characteristic curve (AUC) and true skill statistics (TSS)^91^. To estimate the potential distribution of oak species, the resulting predictions (i.e., probability of species presences) under BART were converted to binary predictions (presence-absence maps) using TSS-maximization thresholds^21,37^.

To obtain assemblage composition and species richness predictions we applied three procedures for stacking species distribution models (S-SDM) (i.e., probability, binary and constrained binary). These procedures return the predicted species composition and richness within each assemblage (i.e., grid cell) across a geographical domain^13,14^. More specifically, probability S-SDM (pS-SDM) and binary S-SDM (bS-SDM) were obtained by stacking the probability of species presence and the binary prediction layers, respectively. Constrained binary S-SDM (cS-SDM) predictions were obtained by constraining each assemblage applying a probability ranking rule (PRR). PRR emulates ecological assembly rules by ranking the species in each assemblage based on the occurrence probability obtained from each species and the number of species per assemblage. The species with the highest probabilities in an assemblage is selected until the number of species in an assemblage, based on observed data, is reached^19,37^. We used the maximum number of species per assemblage from the NEON dataset as the assemblage-level constraint for cS-SDM estimations.

### Evaluating species and assemblage predictions

To evaluate the performance of species assemblage predictions, we used four different metrics that are commonly used for this purpose^20,21,37^. The metrics used were: (a) the deviation of the predicted species richness to the observed (SR deviation); (b) species richness change (SR change); (c) the proportion of correctly predicted as present or absent (Prediction success); (d) true skill statistic (TSS) and (e) similarity between the observed and predicted community composition (Sørensen index). Assemblage metric evaluations were performed using modified functions from the R package ecospat^92^ using the matrices of the three assemblage predictions, i.e., probability, binary and PRR, against the observed assemblage composition from NEON. Note that the SR deviation and SR change were estimated only for the pS-SDM and bS-SDM predictions given that we used the NEON dataset in constraining the number of species per assemblage in the cS-SDM construction.

We also investigated the phylogenetic structure of oak assemblages for both the NEON dataset and the predicted assemblages, in order to explore the performance of predicted oak assemblages in recovering similar patterns of phylogenetic structure as the observed assemblages. To do so, we first obtained the latest phylogenetic hypothesis for oak lineages^93^. This phylogeny was constructed using restriction-site associated DNA sequencing (RAD-seq) and fossil data for node calibration and represent most highly resolved phylogenetic hypothesis for the clade globally^93^. We defined phylogenetic structure as the mean phylogenetic distance (MPD)^94^. To facilitate comparison between the two datasets, we summarized the results using standardized effects sizes (SES), which compare the observed value of an assemblage (MPD) to the mean expected value under a null model, correcting for their standard deviation. SES values > 0 and < 0 indicate phylogenetic clustering and overdispersion, respectively^48,94^. In SES calculations we randomized the tips of the phylogeny to generate random communities (taxon shuffle null model). All phylogenetic structure calculations were conducted using customized scripts and core functions from the picante^95^ package in R.

### Statistical analysis

Using a Bayesian counterpart of Pearson’s correlation test, we evaluated the relationship between species richness and phylogenetic assemblage structure obtained from both NEON and predicted datasets. We chose this Bayesian alternative because it allows robust parameter estimations and accounts for outliers in the data^96^. We also used Bayesian-ANOVAs to test for differences in species assemblage predictions between different modelling procedures (i.e., stacking procedures), using plots as a random variable to correct for potential repeated measures. Using Maximum A Posteriori (MAP) p-values^97^, we then evaluated the evidence for those differences. Note that all analyses were performed for each scale separately, i.e., plot and site scales. Both Bayesian-ANOVAs and the robust Bayesian Pearson’s correlations were implemented in the probabilistic programming language Stan^98^ through the R packages rstanarm^99^ and brms^100^, respectively. All analyses were performed using 4 sampling chains for 10,000 generations and discarding 20% as burn-ins. MAP-based p-values was estimated as implemented in the R package bayestestR^101^.

## Supplementary Information

Supplementary Table S1.

Supplementary Table S2.

## Data Availability

All data used in this paper are already published or publicly available. Data precipitation (CHIRPS) can be obtained from the Climate Hazards group Infrared Precipitation with Stations (CHIRPS-v2—https://www.chc.ucsb.edu/data/chirps) and Land Surface Temperature and Emissivity (LSTE) can be downloaded from EarthData (MOD11C3—https://lpdaac.usgs.gov/products/mod11c3v006/). Predicted oak species models can be found at https://doi.org/10.5281/zenodo.4611525. Code used for modeling oak species distributions is available at https://github.com/jesusNPL/BayesianSDMs_Oaks.
